# Exploring Public Discussions Regarding COVID-19 Vaccinations on Microblogs in China: Findings from Machine Learning Algorithms

**DOI:** 10.3390/ijerph192013476

**Published:** 2022-10-18

**Authors:** Qiong Dang, Shixian Li

**Affiliations:** Climate and Health Communication Center, School of Journalism and Communication, Guangxi University, Nanning 530004, China

**Keywords:** the public, COVID-19 vaccination, sentiment analysis, topic analysis, microblogs, China

## Abstract

Large-scale, widespread COVID-19 vaccination is the most effective means of cutting off the spread of the novel coronavirus and establishing an immune barrier. Due to the large population base in China, it has been a very difficult task to establish such an immune barrier. Therefore, this study aims to explore the public’s discussions related to COVID-19 vaccinations on microblogs and to detect their sentiments toward COVID-19 vaccination so as to improve the vaccination rate in China. This study employed machine learning methods in the field of artificial intelligence to analyze mass data obtained from SinaWeibo. A total of 1,478,875 valid microblog texts were collected between December 2020 and June 2022, the results of which indicated that: (1) overall, negative texts (38.7%) slightly outweighed positive texts (36.1%); “Good” (63%) dominated positive texts, while “disgust” (44.6%) and “fear” (35.8%) dominated negative texts; (2) six overarching themes related to COVID-19 vaccination were identified: public trust in the Chinese government, changes in daily work and study, vaccine economy, international COVID-19 vaccination, the COVID-19 vaccine’s R&D, and COVID-19 vaccination for special groups. These themes and sentiments can clarify the public’s reactions to COVID-19 vaccination and help Chinese officials’ response to vaccine hesitancy. Furthermore, this study seeks to make up for the lack of focus on big data in public health and epidemiology research, and to provide novel insights for future studies.

## 1. Introduction

Following the outbreak of the coronavirus disease (COVID-19) in Wuhan, China, it quickly developed into a global crisis that has caused immeasurable damage to people’s lives, property, health, and their economic and social development [1,2,3,4]. Studies have demonstrated that vaccination is the most effective means to prevent and control COVID-19 [5,6], and thus to establish an immunological barrier. Therefore, the National Health Commission of China has strongly recommended all-staff vaccinations against COVID-19. Subsequently, several policies, such as the Technical Guidelines for Novel Coronavirus Inoculation (First Edition) [7], the Notice of Central Financial Subsidy Funds for New Coronavirus Vaccines and Inoculation Costs in 2021 [8], and the Technical Guidelines for Vaccination of New Coronavirus (First Edition) [9], were issued to guide and encourage people to get vaccinated against COVID-19. In line with this, domestic vaccines such as the Sinovac, Sinopharm, and CanSino vaccines were approved for use in vaccinations. As such, the Chinese government played an absolutely dominant role in the Chinese COVID-19 vaccination drive. However, the responses, emotions, and perceptions of the public in China to COVID-19 vaccination have been ignored, and therefore need to be further explored, given their great significance to taking effective measures to reduce vaccine hesitancy, and thus to accelerate the establishment of immunological barriers nationwide.

Social media such as Twitter, Sina Weibo and Facebook/Meta are platforms for free expression [10,11,12] and can provide extensive and valuable information to explore users’ awareness and sentiments on major public health events such as COVID-19 vaccinations [13,14]. Moreover, the analysis of social media data has become one of the most important fields of focus in medical informatics research. In the current literature, several studies have been conducted to examine topics related to COVID-19 using social media data. For example, Cotfas et al. [15] used Twitter data to study the dynamics of COVID-19 vaccination opinions in the UK, while Hussain et al. [16] compared public sentiment and attitudes towards the new crown vaccines in the UK and US using data from Facebook and Twitter. In addition, Liu et al. [17] probed the factors influencing propensity toward COVID-19 vaccination with Twitter data. It is worth noting here that most of the research data concerning public awareness and sentiments for vaccination come from Twitter and Facebook, and little research has been conducted based on data from microblogs in China.

To address the gap, the current study employed the machine learning method to explore public discussions concerning COVID-19 vaccination on Sina Weibo in China. The study’s objectives were twofold: (1) to excavate Chinese public sentiments associated with COVID-19 vaccination (RO1), and (2) to identify the themes that the Chinese public discussed concerning COVID-19 vaccination (RO2). This study was conducted in two main steps in the Chinese context: first, data collection was performed using Python code to crawl the texts related to COVID-19 vaccination on the Sina Weibo platform, and then data analysis was used to confirm trends, keywords, sentiments, and themes concerning COVID-19 vaccination on microblogs in China.

## 2. Materials and Methods

### 2.1. Data Collection Procedure

Sina Weibo is a Chinese microblogging (weibo) website. It is one of the most popular social media platforms, where the public can obtain the latest health information and freely express their opinions on current affairs in China [18]. By 31 March, 2022, the active monthly users of Sina Weibo platform had reached 582 million, and the daily active users had reached 252 million [19]. In addition, data collected from the microblog platforms are acceptable for research, being extremely abundant, valuable, and useful [20,21].

**Figure 1:**
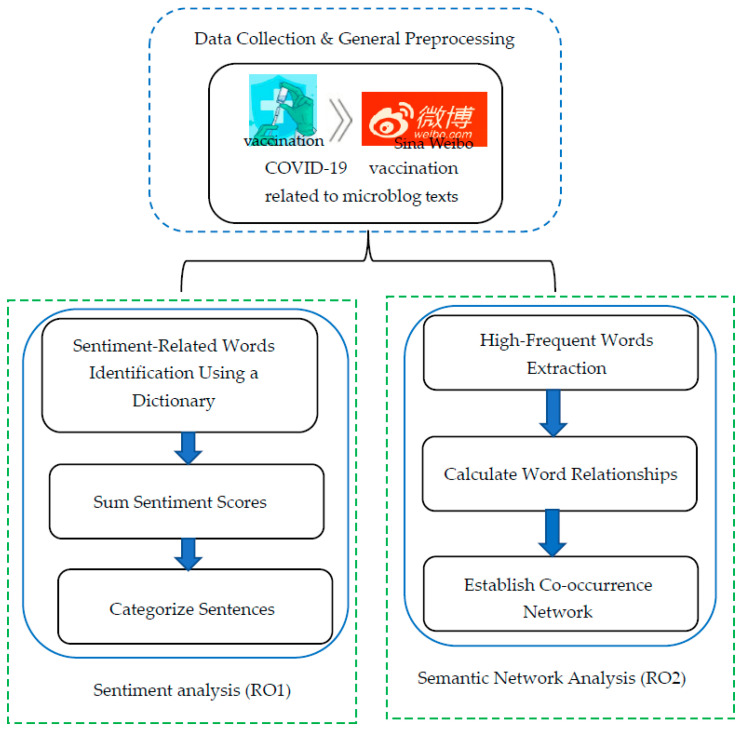
Data processing workflow in the current study.

Therefore, the current study used Python self-compiled code (the code is available on request) to crawl data concerning COVID-19 vaccination from China’s Sina Weibo platforms. The keywords were set as “COVID-19 vaccination”, “novel coronavirus vaccination”, “COVID-19 vaccine”, “novel coronavirus vaccine”, and “COVID-19 vaccine inoculation”. The language was set as Chinese. The time range was set from 15 December 2020 (an important date when China officially launched their vaccination campaign for key groups) to 30 June 2022. A total of 1,835,007 texts related to COVID-19 vaccination were gathered. Each collected text included the date/time, text, username, number of retweets, likes, quotes, and replies. To ensure the validity of the collected data, those texts which had a total number of forwards, comments, and likes greater than or equal to 3 were retained. Moreover, in order to avoid causing any confusion, we also manually checked and verified all the collected data. Finally, a total of 1,478,875 valid texts related to COVID-19 vaccination were retained for further analysis. The data processing workflow was presented in Figure 1. The full sample data is available on request.

### 2.2. Research Methods

#### 2.2.1. Sentiment Analysis

Sentiment analysis, as a machine learning technique, is used to detect the positive, negative, or neutral sentiments expressed in a text [22]. It is typically used to analyze the content of web-based texts [11,16,17], and has been increasingly popular in the field of public health and preventive medicine [23,24,25,26,27]. Thus far, there are several sentiment dictionaries such as the English NRC sentiment dictionary and Chinese Emotional Vocabulary Ontology Database of the Dalian University of Technology that have been widely used to uncover sentiments expressed in web texts [28,29,30,31]. Significantly, although the English dictionary supports Chinese, it is hard to fully adapt it to the Chinese context due to production issues [32]. Therefore, to ensure the reliability and validity of the results, this study selected the Chinese Emotional Vocabulary Ontology Database of the Dalian University of Technology to analyze the sentiments contained in microblog texts related to COVID-19 vaccination. The Chinese Emotional Vocabulary Ontology Database was developed by Li Hongfei, a professor at Dalian University of Technology, China and his team, including 27,467 words [33]. These sentiment words were divided into three categories: neutral, positive, or negative, corresponding to the values of 0, 1, and 2, respectively. Moreover, the polarity intensity of the sentiment vocabulary was set to five levels, 1, 3, 5, 7, and 9. Among them, 9 represents the maximum intensity, while 1 represents the minimum intensity. Drawing on Ekman’s six basic emotions theory [34,35], the Chinese dictionary classified emotions into 7 major categories (anger, disgust, fear, sadness, surprise, wellness, and happiness) and 21 minor categories.

The analysis procedure was as follows. First, we read the collected microblog data using Pandas (the Python data analysis library), and then imported them into the Chinese Emotional Vocabulary Ontology Database; we then ascertained the distribution of the seven emotions in the collected microblog data. The calculation rules for the values of positive and negative sentiments were as follows: positive = happy + good + surprise and negative = anger + sadness + fear + disgust. In addition, we incorporated the Chinese word segmentation dictionary and custom stop dictionary in order to filter nonsense stop words, and to subsequently calculate the frequency of the occurrence of the seven emotional eigenvalues. Finally, we drew a sentiment word cloud map for COVID-19 vaccination with WordCloud in PyEcharts.

#### 2.2.2. Semantic Network Analysis

Semantic network analysis is a form of knowledge expression and has been widely used in artificial intelligence [36,37,38]. It is also one of the most commonly used content analysis methods, and is characterized by taking high-frequency words as nodes, reflecting the relationship among the nodes via the co-occurrence times of high-frequency word combinations, analyzing the semantics of high-frequency word combinations in the text by constructing a semantic network, and ultimately presenting the mental map of information in the form of a directed graph [36]. Therefore, this study used semantic network analysis to extract and summarize the themes, characteristics, and inherent tendencies of microblog texts related to COVID-19 vaccination.

We used the Jieba library in Python to segment the Chinese sentences in the collected microblog texts. Specifically, the Jieba library is currently the most commonly used library in Chinese word segmentation [39,40,41]. Before this, word cleaning had been performed, including noise data filtering, stop words processing, and synonym merging. Then, we used pointwise mutual information (PMI) to calculate the similarity of all the words and to then extract the 300 words with the highest co-occurrence frequency. In particular, PMI is a quantitative and systematic approach that measures the correlation between two items and is also an unsupervised machine learning method suitable for performing topic modeling [42]. PMI utilizes the similarity between words to build a word relationship network, and to then carry out a thematic clustering analysis [43,44,45]. The PMI formula is as follows:

$$PMI\left(x,y\right)=\log_{2}\frac{p\left(x,y\right)}{p\left(x\right)p\left(y\right)}$$

*x* and *y* are words or sets of words, *p* (*x*, *y*) is the probability that they co-occur, and *p*(*x*) and *p*(*y*) are the probabilities of *x* and *y* occurring in the corpus, respectively. PMI will be largely positive if *x* and *y* are strongly associated, or highly negative if they are complementary, and near zero if there is no significant relationship between them. Finally, the top 300 words were imported into Gephi for visual analysis, and after the modularization operation, six clustered topics had been identified.

## 3. Results

### 3.1. Descriptive Analysis of Microblog Texts Related to COVID-19 Vaccination

**Figure 2:**
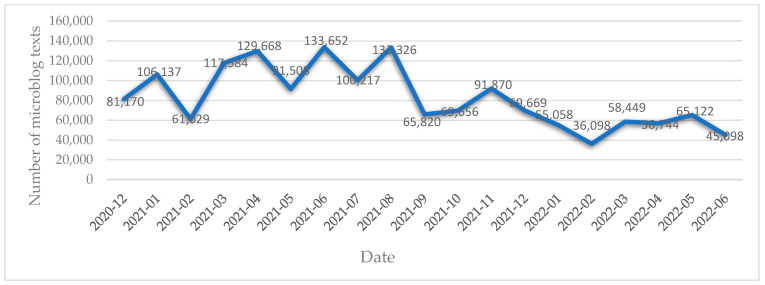
Number of Microblogs entries related to COVID-19 vaccination from December 2020 to June 2022.

A total of 1,478,875 valid microblog texts related to COVID-19 vaccination were retrieved from more than 510,000 microblog users, representing 660 cities within the 31 provinces of China. Figure 2 displays the number of microblog texts related to COVID-19 vaccination from December 2020 to June 2022. The discussion on COVID-19 vaccination can be divided into three stages: the beginning stage (December 2020–February 2021), the fluctuating increase stage (March 2021–August 2021), and the decreasing stage (September 2021–June 2022). On the whole, there was a gradual decline in the number of microblog texts over time, from which a decreasing zeal in discussing COVID-19 vaccination can be inferred.

In the beginning stage, the number of microblog texts related to COVID-19 remained relatively small with a total of 248,334 texts, representing the smallest proportion (16.8%). It is worth noting there is a sharp increase observed at the end of December 2020, the possible reason for which could be that this is when the Joint Prevention and Control Mechanism of the State Council of China announced that the inactivated COVID-19 vaccine produced by the China National Pharmaceutical Group Corporation had been approved by the State Food and Drug Administration for conditional listing, and that all people could be vaccinated free of charge from 31 December 2020. The news that China’s first COVID-19 vaccine was on the market indeed injected confidence into people’s fight against the pandemic, which triggered extensive discussions on microblogs. In the second stage, the number of microblog texts increased in a horizontal S-shape curve, accounting for 43.7% of the total (the largest proportion). One notable feature is that ordinary people begin to get vaccinated against COVID-19, indicating that they may have actively been expressing their feelings on microblogs after having been vaccinated. In this latter stage, the number of microblog texts stood at 39.5% of the total, with the quantity of texts reaching a minimum of 36,098 in February 2022.

### 3.2. Identify Sentiments in Microblog Texts Related to COVID-19 Vaccination

The results of the sentiment analysis identified that 36.1% microblog texts conveyed positive sentiments on COVID-19 vaccination, among which surprise accounted for 3.5%, good stood at 63% and happiness was 3.5%, while 38.7% of the microblog texts expressed negative sentiments on COVID-19 vaccination, among which anger, disgust, fear, and sadness accounted for 2.9%, 44.6%, 35.8%, and 16.7% of the total negative sentiments, respectively. Moreover, 25.2% of the microblog texts held neutral sentiments on COVID-19 vaccination. Overall, microblog texts with negative sentiments slightly outweighed those with positive emotions.

**Figure 3:**
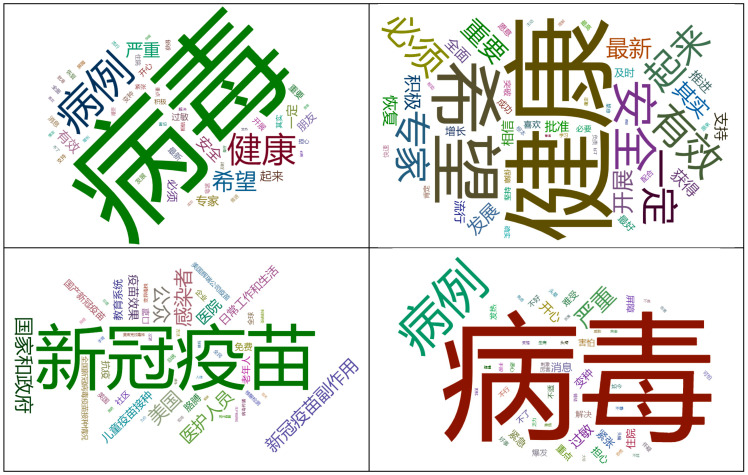
Word clouds containing the 50 most frequently used words related to the COVID-19 vaccination on microblog platforms. The upper left image is a word cloud formed from all microblog texts, while the upper right image is formed from microblog texts incorporating positive sentiments. The lower left image is formed from microblog texts of neutral sentiment, while the lower right image is formed from microblog texts of negative sentiment.

The most positive sentiment words consisted of “health”, “hope”, “security”, “effect”, “recovery”, “support”, “succeed”, and “breakthrough”, whereas the most negative sentiment words contain “virus”, “COVID-19 cases”, “serious”, “allergy”, “variation”, “hospitalization”, “worry”, “fear”, and “erupt” (Figure 3 and Table 1). Examples of microblogs expressing positive, neutral, and negative sentiments are displayed in Table 2.

**Figure 4:**
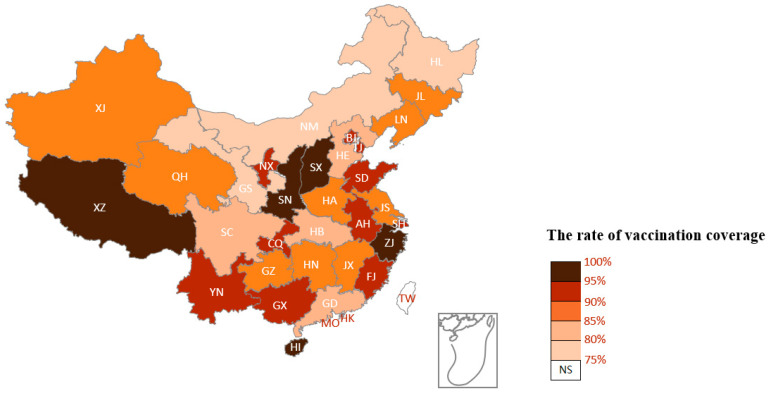
Distribution of COVID-19 vaccination rate in each province of mainland China.

Figure 4 displays a distribution of the COVID-19 vaccination rate in each province in China, aiming to reveal the public’ sentiment in different provinces on COVID-19 vaccination from a macro perspective. The darker the color of a certain province is, the higher the vaccination rate there. Correspondingly, the lighter colors illustrate that the vaccination rate is lower. To a large extent, the higher the vaccination rate, the more positive the public’s attitude towards vaccination [46]. Among the 31 provinces, Tibet, Zhengjiang, Shanxi, Shan’xi, and Hainan represent the highest vaccination rates, indicating that the public in these regions hold more positive sentiments on vaccination. Nei Monggol, Gansu, and Heilongjiang represent the lowest vaccination rates, revealing that people here harbor more negative sentiments on vaccination.

### 3.3. Identify Latent Themes

**Figure 5:**
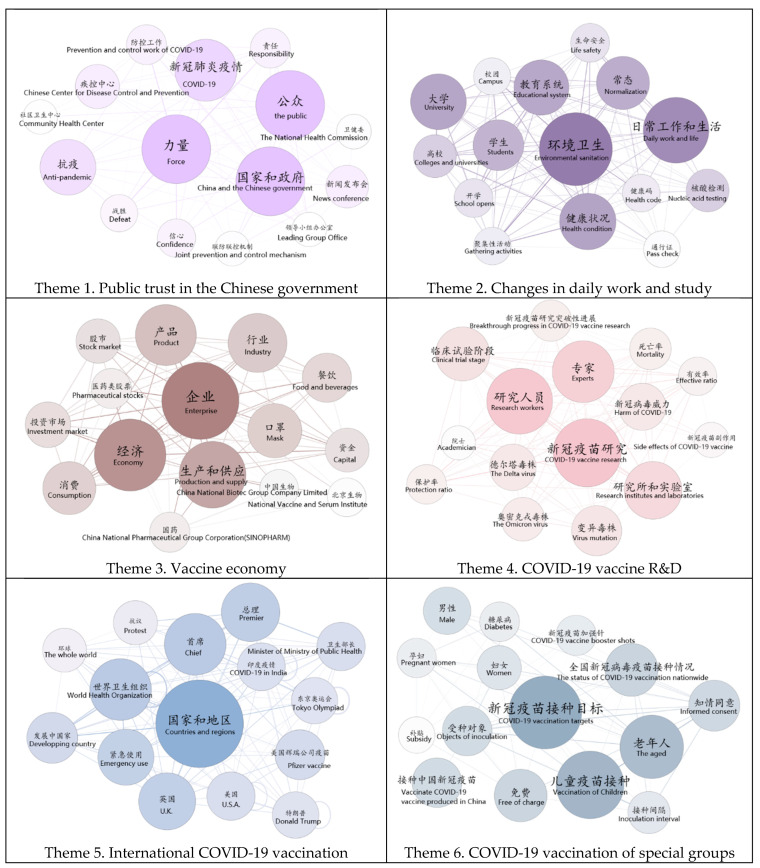
Semantic network graphs of six themes concerning COVID-19 vaccination on microblog platforms in China, with the top 15 associated words per topic (note: the authors added the English translations for a better understanding).

The results illustrated that six salient topics dominated discussions on COVID-19 vaccination held on microblogs, and thus each of the six topics was labeled with a theme: public trust in the Chinese government (theme 1), changes in daily work and study (theme 2), vaccine economy (theme 3), international COVID-19 vaccination (theme 4), COVID-19 vaccine research and development (R&D) (theme 5), and the COVID-19 vaccination of special groups (theme 6). Figure 5 displays six semantic network graphs, each corresponding to one of the six themes. Each graph contains the 15 most frequently used words in microblog texts corresponding to a specific theme.

Each node in the graph represents a high-frequency word. The larger the size of the node, the higher the frequency of the node; that is, the higher the importance of the word. The lines between the words are referred to as links or actions, and these are used to illustrate the relationships between nodes [47,48]. Specifically, the thicker the connection, the closer the relationship is. “The public”, “China and the Chinese government”, and “force” dominated the discussions of Theme 1, while “environmental sanitation”, “nucleic acid testing”, and “educational system” dominated the discussions of Theme 2. For vaccine economy, “enterprise”, “economy”, and “production and supply” dominated, while for international vaccination, “countries and regions”, “World Health Organization”, and “US”, “UK” and “COVID-19 in India” dominated. Regarding the R&D of COVID-19 vaccines, “COVID-19 vaccine research” “researchers”, and “experts” dominated. In Theme 6, “COVID-19 vaccination targets”, “vaccination of children”, and “the aged” dominated.

## 4. Discussion

In China, the current situation surrounding the prevention and control of the COVID-19 pandemic is still severe and complex [49], but vaccination against the novel coronavirus remains the most effective means. It is an inevitable trend to actively inoculate the population with the COVID-19 vaccine to build a nationwide immune barrier. As such, this study explores the public’s discussions on COVID-19 vaccination in China.

### 4.1. Sentiments

Sentiment analysis is useful for capturing the public’s perception of an event [13,17]. The results of this study’s sentiment analysis indicated that the positive sentiments include good, happiness, and surprise, while the negative sentiments contain anger, disgust, fear, and sadness. Overall, although the sentiments expressed in microblog texts related to COVID-19 vaccination were more likely to be negative, the positive sentiment texts strongly indicated that the public in China has high expectations that vaccinations can effectively prevent COVID-19. Significantly, negative sentiment about COVID-19 vaccination was more prevalent in sparsely populated and economically underdeveloped provinces with lower infection rates, which is consistent with the study of Huang et al. [14].

Keywords with a positive sentiment commonly expressed gratitude for the motherland to provide such an opportunity to get vaccinated for free, implying that as a socialist country, the Chinese government has strong credibility and executive power in the face of major public crisis event. In addition, these keywords also expressed the public’s trust in the safety and effectiveness of the COVID-19 vaccine, although a few keywords conveyed negative sentiments on imported COVID-19 vaccines, thereby unmasking their ambivalence about such imported vaccines. Some of the terms used compared COVID-19 vaccines to weapons that can destroy an enemy with the aim of maintaining social stability. Another form of positive sentiment was the hope that the vaccines offered to the infected through “recovery” and “healthy” trends. The high proportion of positive sentiments suggests that the majority of Chinese have a basic understanding of COVID-19 vaccinations, and a cautionary note is that economically-developed provinces such as Zhejiang or provinces with friendly policy governance such as Tibet had a higher predominance of positive sentiment on COVID-19 vaccination.

Negative sentiments of the public on COVID-19 vaccination were complicated, and mainly included anger, disgust, fear, and sadness. A common negative emotion was fear that the side effects of vaccination could lead to death, implying that COVID-19 vaccines, such as those of Pfizer and Sinovac, may have some inherent “defects”. Furthermore, additional negative sentiment keywords expressed outrage that officials were prioritizing vaccinations of the public over front-line workers in certain places, while other keywords indicated anxiety about whether people with diabetes, depression, anxiety, or neuroses can be vaccinated against COVID-19. In addition, other keywords have also expressed distaste for government groups in other countries using COVID-19 vaccines to manipulate the public and serve their political interests.

Significantly, the most prominent negative sentiments concerned doubts and complaints from the public about the need for mandatory COVID-19 vaccinations in China. The reason for this may be that National Health Commission of China has emphasized that COVID-19 vaccination follows the basic principles of “informed, consensual, and voluntary”, and that people should not be forced to be vaccinated [50]. However, in reality, the communities and units hinted that individuals will be punished if they do not get vaccinated, which illustrates that the policy has been shelved or distorted, thus triggering public resentment. It can be further predicted that these negative sentiments provoked more intense discussions on injustice, abuse of power, and inequality surrounding the COVID-19 vaccination drive, which may have led to the sharing of a large amount of false information on social media platforms and thus misleading people. Targeted measures should be taken to ease the public’s dissent so as to properly guide public opinion in the correct direction.

### 4.2. Microblog Discussion Themes Related to COVID-19 Vaccinations in China

#### 4.2.1. Public Trust in the Chinese Government

The results indicated that discussions surrounding the public’s trust in the Chinese government usually overlapped with politics. For example, most microblog texts placed particular stress on the important role of the government’s policy support and financial investment in COVID-19 vaccine R&D, and believed that Chinese-manufactured vaccines are safe, effective, and high quality [51,52,53]. Moreover, some discussions expressed their approval concerning the COVID-19 vaccination strategies, such as free all-staff vaccinations, medical insurance, and financial subsidies [54,55], indicating that the Chinese government regards people’s health as its top priority. In addition, the public discussed the issue that the Chinese government donated vaccines and provided vaccine assistance to other developing countries, such as Pakistan, Cambodia, and Laos [56], and considered that these actions facilitated global vaccine equity. Ultimately, these findings tended to correspond to the public’s attitude and the government’s governing ability, and stressed the significant role of China in major public emergencies.

#### 4.2.2. Changes in Daily Work and Study

Large-scale vaccination is the most effective means of halting the spread of the virus and establishing an immune barrier [57,58]. Discussions dealing with this focused on the keywords “environmental sanitation”, with links to “health condition”, “daily work and life”, and the “education system”. Changes in people’s daily lives mainly involved them having to share their “vaccine passports” or certificates before entering public places, such as their place of work, restaurants, cinemas, gyms, schools, and hospitals. A prominent change was that students were required to be vaccinated against COVID-19 and to learn vaccine-related knowledge in order to reduce the risk of cluster infections in the educational environment.

#### 4.2.3. Vaccine Economy

When a vaccine becomes a commodity and is produced, circulated, sold, and distributed on a large scale in society, it has social and economic attributes [59]. The study found that the “COVID-19 vaccine” was an overwhelming topic of discussion and was definitively linked to “enterprise”, “economy”, “production and supply”, the “China Nation Biotec Group Campy Limited”, and “pharmaceutical stocks”, indicating that the economic effects of the COVID-19 vaccine triggered widespread discussions. In these discussions, some displayed their strong enthusiasm towards buying concept stocks concerning vaccines and in exploring the international vaccine trade [60,61]. However, some criticized this so-called “vaccine economy” because they considered vaccine products to have a strong public utility attribute [62] and that the government’s participation in procurement is significantly higher than that in other pharmaceutical fields [63]. Overall, these findings illustrated that discussions surrounding the vaccine economy may be beneficial to streamlining the resource-input related to vaccine production and distribution, as well as the utility and benefits generated, which effectively identify the optimal allocation of vaccine-related health resources in society, thereby assisting the development of the vaccine industry in China.

#### 4.2.4. COVID-19 Vaccine R&D

COVID-19 vaccine R&D has been used to combat the global COVID-19 pandemic and to curb the deterioration of the pandemic. Overwhelmingly, discussions focused on the term “COVID-19 vaccine research”, with links to “research workers”, “experts”, and “research institutes and laboratories”. Another interesting finding is that discussants expressed their reverence for researchers and experts who have made significant contributions to COVID-19 vaccine R&D, such as Zhong Nanshan, Zhang Wenhong, and Wu Zunyou [64,65,66]. Moreover, some discussions were centered around “COVID-19 vaccine R&D”, highly overlapping with “Delta virus”, “Omicron virus”, “mutation”, and “mortality” [67,68], revealing concerns regarding the difficulties of vaccine R&D caused by the virus’ mutation. However, there are some voices against vaccine R&D because they claim vaccines are useless, harmful, and a sheer waste of energy and money [69]. Overall, the public held conflicting opinions on vaccine R&D, but most discussions believe that vaccine R&D is utterly valuable and requisite.

#### 4.2.5. International COVID-19 Vaccination

Discussions surrounding international COVID-19 vaccination drives are usually inextricably linked to culture and politics in different countries. The majority of these discussions have centered around “countries and regions”, with strong links to “the United States”, “emergency use”, “the United Kingdom”, “the World Health Organization”, and “COVID-19 in India”. More specifically, the current study found that most microblog texts held a relatively objective opinion on the COVID-19 vaccination campaign in the US. On the one hand, the public considered that vaccines produced in the US were safe and effective [70,71]; on the other hand, they criticized a series of socio-political issues triggered by COVID-19 vaccinations there, mainly including vaccine racism and nationalism, and the degradation of the government’s credibility [72,73,74]. Moreover, most microblog texts discussed the US presidential elections and Trump and Biden’s vaccination policies [75,76]. Among them, the topic of “Biden’s live vaccination” has been widely shared. These indicated that vaccinations have been heavily politicized in the US. Furthermore, the topics concerning vaccination in the UK, such as the international trade in vaccines, the effects of COVID-19 mutations on the market, the campaign against vaccination, and the vaccinations of the Queen of the UK were extensively discussed. In addition, vaccinations in India also attracted widescale attention. One hot topic was that the British government did not recognize the “COVID-19 vaccine certificate” issued by the Indian authorities, which has been criticized by India as a “discriminatory policy” [77], and one which deviates from the declaration of Secretary General of the United Nations “to maintain vaccine fairness is to protect human rights, and to condone vaccine nationalism is to disregard human rights” [78].

#### 4.2.6. The COVID-19 Vaccination of Special Groups

The vaccination strategy in China started with high-risk groups and other key groups, and then gradually transitioned to children and the elderly over 60 years old [79]. This study found that “COVID-19 vaccination targets” was an overwhelming topic of discussion and was definitely linked to “children’s vaccination”, “free of charge”, “the aged”, and “objects of inoculation”, indicating that minors and the aged are the important groups for vaccination with the final purpose of building a barrier representing herd immunity in China. However, most discussions laid particular emphasis on parents’ worries about their vaccination of their children, while some of the discussants questioned the preposterous school regulations dictating that children cannot go to school without being vaccinated against COVID-19. Another hot topic has been whether the vaccine would cause high mortality when the elderly were vaccinated. This theme became a hot topic because China adopted the “zero-case” policy to minimize mortality and infection rates.

On the whole, although vaccination itself is an objective concern, it is inextricably linked with the politics, economy, and culture of China. Specifically, the Chinese government’s vaccine policy and negative social events caused by vaccination were the main subjects of discussion. The vaccine has economic attributes and the economic effects of the COVID-19 vaccine triggered widespread discussions. Moreover, the social issues triggered by such vaccination campaigns are more likely to be widely discussed on microblogs.

## 5. Potential Impact

Our results illustrated that the application of machine learning methods for mining Sina Weibo texts related to COVID-19 vaccination can yield valuable and useful data for public health departments, governments, policymakers, and health communication researchers. For example, we found that the fear of vaccine side effects and misleading information are important causes of vaccine hesitancy. Therefore, Chinese public health departments should make appropriate strategies and engage in effective communication. Moreover, policy makers should advocate for the establishment of national surveillance systems to oversee and censor internet-based content, especially social media, in order to better understand the sentiments of the public and to mitigate the impact of these negative sentiments towards vaccination. Additionally, it is worth noting that the elderly and children are special groups for vaccination in China. Therefore, policymakers should formulate technical guidelines and compensatory measures for COVID-19 vaccination for them in order to alleviate parents’ concerns about children’s vaccination and to guide and encourage the elderly to get vaccinated to protect their health and interests when the safety of vaccines may be in question. As traditional public health data cannot become available in time, therefore, digital data such as microblog data has become extremely crucial, and when such data is appropriate analyzed, it can enrich epidemiologic data in real-time so as to conduct a more all-round and instantaneous assessment of the COVID-19 vaccination.

In addition, microblogs provide an inexpensive and efficient platform for assessing the effectiveness of public health communications [20], and thus for targeting public health campaigns based on the dominant topics of microblog discussions. For example, microblog text analysis regarding the side effects of vaccination can evaluate the effects of information transmission. Mining microblog data can also provide certain insights into how the public interprets complex situation concerning international vaccination, such as vaccination policies in different countries and inequality in global vaccination drives. Moreover, herd immunity can only be achieved with high vaccination rates [80]. However, unsubstantiated concerns about the negative side effects of vaccines may overshadow the benefits of vaccination. Microblogs offer an opportunity to focus on vaccine acceptance and to tailor responses to those who oppose vaccination. People refusing vaccination gather together on social media platforms to express more negative sentiments about vaccination, which can amplify the risk that vaccination can prevent diseases. Microblog text analysis provides public health officials a potentially powerful and inexpensive tool to track these people and to then take interventional measures.

Compared to traditional research methods, such as questionnaires and interviews, machine learning has the ability to deal with multi-variate data in a short time and to extract hidden relationships within massive data-sets in a dynamic, complex, and even chaotic environment [81,82], as is the case the environment of public health emergencies. Moreover, since most public health and epidemiological problems require rich data for referencing, machine learning offers researchers and practitioners a tool by which to increase their understanding of these fields.

## 6. Conclusions, Limitations, and Future Research

### 6.1. Conclusions

Microblog data analysis can be used to explain the public’s awareness and perception of the COVID-19 vaccination in China. The current study aims (1) to excavate Chinese public sentiments associated with COVID-19 vaccination and (2) to identify the themes that the Chinese public discussed concerning COVID-19 vaccination. We found that discussions dealing with COVID-19 vaccinations on Sina Weibo could be divided into three main stages: the beginning stage, the fluctuating increase stage, and the decreasing stage. Overall, the number of discussions has gradually declined over time.

In terms of sentiment analysis, negative microblog texts slightly outweighed positive microblog texts. Positive sentiments included surprise, good, and happiness, among which good dominated the positive microblog texts, while negative sentiments contained anger, disgust, fear, sadness, among which disgust and fear were predominant. These sentiments can discern the public’s reactions to COVID-19 vaccination and thus help public health departments, government officials, and medical experts to take effective measures to improve the vaccination rate and to accelerate the establishment of immune barriers nationwide.

With regard to semantic network analysis, our study confirmed six overarching themes related to COVID-19 vaccination: public trust in the Chinese government, changes in daily work and study, the vaccine economy, international COVID-19 vaccination, COVID-19 vaccine R&D, and the COVID-19 vaccination of special groups.

### 6.2. Limitations and Future Research

It is undeniable that our study has also encountered several limitations. First, more than 90% of microblog users are young and middle-aged people according to the report of Sina Weibo Data Center [83]. Therefore, there is insufficient data on the vaccination attitudes of the elderly, children, and non-microblog users. Future research can therefore combine text mining and questionnaires/surveys to conduct a more comprehensive analysis of the public’s vaccination awareness and sentiments. Furthermore, the application of the machine learning method to the analysis of microblog texts represents a potential weak point since it may not be able to treat data as carefully as humans might [84]. However, machine learning can objectively process mass data in a short time, making it faster and more effective than manual methods. In addition, our model can be used to handle other social platform problems; for example, future research can focus on negative sentiment data from Twitter, Facebook/Meta, YouTube, and TikTok to detect whether false information or rumors exist, so as to provide appropriate suggestions for how to correctly guide the trends in public opinion.

## Figures and Tables

**Table 1:** Top 50 most frequently used words related to COVID-19 vaccination on microblog platforms.

All Microblogs	Positive Sentiment	Negative Sentiment	Neutral Sentiment
Virus	Health	Virus	COVID-19 vaccination
Covid-19 cases	Hope	COVID-19 cases	The novel coronavirus
Health	Security	Serious	COVID-19 vaccine
Hope	Effect	Allergy	Covid-19 vaccine booster shots
Serious	Certain	Unhappy	COVID-19
Security	Stand up	Variation	Infected individuals
Effective	Expert	News	State and government
Certain	Must	Nervous	America
Stand up	Friend	Hospitalization	The public
Expert	Significant	Urgent	Side effects of the COVID-19 vaccine
Must	Up-to-date	Worry	Medical workers
Friend	Carry out	Barrier	Hospital
Allergy	Actually	Key point	Vaccine effectiveness
Significant	Positive	Uncomfortable	Vaccination of children
Up-to-date	Development	Fear	Daily work and life
Happy	Obtain	Handle	Educational system
Improve	Recovery	Without end	Arm
Actually	Support	Erupt	The aged
Variation	All-round	Fever	Anti-epidemic
News	Trust	Bad	COVID-19 vaccine developed and made in China
Positive	Advance	Discomfort	Free of charge
Development	Approve	No way	Community
Nervous	Popular	Deed	Mask
Hospitalization	Increase	Severe	The status of COVID-19 vaccination nationwide
Emergent	Timely	Nowadays	Enterprise
Acquire	Best	Dreadful	Global
Recovery	Like	Suspect	The U.K.
Worry	Succeed	Dizzy	Pfizer vaccine
Support	Breakthrough	Nausea	Mutation
Comprehensive	Requisite	Stimulate	President
Trust	Be willing	Means	The entire people
Protective screen	Guarantee	Feeble	Nucleic acid testing
Keynote	Coordinate	Headache	Virus transmission
Uncomfortable	Persist in	Fall ill	Arm
Advance	Affirm	Insufficient	Wuhan
Approve	Reliable	Mood	Method
Prevalent	Fast	Headache	Omicron virus
Fear	Highest	Panic	Scene
Solve	Fundamental	Disguise	Menstruation
Increase	Responsibility	Anxious	India
Timely	Apply for	Threaten	Human body
Without end	Original	Worry	Scientific research
Best	Study	Lost	Nine valent HPV vaccine
Erupt	Confirm	Hurt	Aunt
Like	Help	Trouble	Sinopharm
Succeed	Active	Rampant	Expert
Fever	Understand	The elderly	Rabid dog
Break through	Ascend	Harmful	Production and supply
Necessary	Ordinary	Hesitate	Sisters
Be willing	Education	Deficiency	Sinovac vaccine

**Table 2:** Examples of microblog texts expressing positive, neutral, and negative sentiments about the COVID-19 vaccination.

Positive Sentiments	Neutral Sentiments	Negative Sentiments
#China’s COVID-19 vaccine was approved for marketing# Great! After vaccination, normal life and production will resume soon. Free vaccination for all! Free vaccination is really great! I am very confident in the safety and effectiveness of the vaccine. I encourage everyone to be vaccinated, so that we can put a protective veil on our country and thus end this pandemic. As the first batch of people in the unit to get COVID-19 vaccine, I went to check the antibody titer a month later, and the results showed that the vaccine worked well. When I was vaccinated, I sincerely thanked the motherland for its strength! The motherland protects us! The Bidens were publicly vaccinated with the COVID-19 vaccine, conveying a message to the public that the vaccination is safe, and the vaccine complies with medical regulations.	We still need to continue to take protective measures after vaccination, including wearing masks, maintaining social distance, washing hands frequently. The doctor advised me not to get the COVID-19 vaccine and HPV vaccine at the same time. After vaccination, there was no adverse reaction except that the deltoid muscle of my left arm was a little sore. China attaches great importance to the safety and effectiveness of COVID-19 vaccine and COVID-19 vaccine R&D. Actively participate in the vaccination of COVID-19 and jointly build the great wall of immunity! (Slogan in universities in Henan province)	I just hate and oppose to the “executors” and “advocates” who openly say that you can choose to be vaccinated voluntarily but secretly take measures to force you to be vaccinated. I have diabetes, so I’m afraid of getting COVID-19 vaccine. I suffer from depression, so I can’t get COVID-19 vaccine, which makes me feel very sad. Why did I still get COVID-19 after vaccinating three doses of the COVID-19 vaccine? I feel so sad. People around me didn’t vaccinate because they were afraid of side effects leading to other major diseases. If I don’t get the COVID-19 vaccine, I can’t participate in the project construction. I am disgusted with the mandatory requirement for the public to be vaccinated against COVID-19.
